# The protective effects of melatonin against electromagnetic waves of cell phones in animal models: A systematic review

**DOI:** 10.1002/ame2.12552

**Published:** 2025-02-24

**Authors:** Mohammad Amiri, Habibolah Khazaie, Masoud Mohammadi

**Affiliations:** ^1^ Sleep Disorders Research Center Kermanshah University of Medical Sciences Kermanshah Iran; ^2^ Research Center for Social Determinants of Health Jahrom University of Medical Sciences Jahrom Iran

**Keywords:** animal model, cell phone radiation, melatonin

## Abstract

**Background:**

Due to the widespread use of cell phone devices today, numerous research studies have focused on the adverse effects of electromagnetic radiation on human neuropsychological and reproductive systems. In most studies, oxidative stress has been identified as the primary pathophysiological mechanism underlying the harmful effects of electromagnetic waves. This paper aims to provide a holistic review of the protective effects of melatonin against cell phone‐induced electromagnetic waves on various organs.

**Methods:**

This study is a systematic review of articles chosen by searching Google Scholar, PubMed, Embase, Scopus, Web of Science, and Science Direct using the keywords ‘melatonin’, ‘cell phone radiation’, and ‘animal model’. The search focused on articles written in English, which were reviewed and evaluated. The PRISMA process was used to review the articles chosen for the study, and the JBI checklist was used to check the quality of the reviewed articles.

**Results:**

In the final review of 11 valid quality‐checked articles, the effects of melatonin in the intervention group, the effects of electromagnetic waves in the case group, and the amount of melatonin in the chosen organ, i.e. brain, skin, eyes, testis and the kidney were thoroughly examined. The review showed that electromagnetic waves increase cellular anti‐oxidative activity in different tissues such as the brain, the skin, the eyes, the testis, and the kidneys. Melatonin can considerably augment the anti‐oxidative system of cells and protect tissues; these measurements were significantly increased in control groups. Electromagnetic waves can induce tissue atrophy and cell death in various organs including the brain and the skin and this effect was highly decreased by melatonin.

**Conclusion:**

Our review confirms that melatonin effectively protects the organs of animal models against electromagnetic waves. In light of this conclusion and the current world‐wide use of melatonin, future studies should advance to the stages of human clinical trials. We also recommend that more research in the field of melatonin physiology is conducted in order to protect exposed cells from dying and that melatonin should be considered as a pharmaceutical option for treating the complications resulting from electromagnetic waves in humans.

## Graphical abstract

Systematic process of collecting studies based on PRISMA.

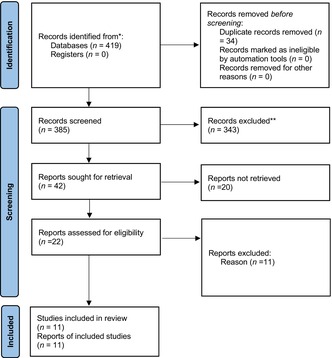

## 1 BACKGROUND

Nowadays, the extensive usage of mobile phones and other electronic devices that produce magnetic rays has raised increasing concern among the scientific community and the general public regarding the destructive impacts of these rays. Although electromagnetic hypersensitivity (EHS) has not yet been formally recognized by the World Health Organization, there have been various studies on the harmful impacts of this phenomenon on neuropsychological systems.^1^

In these studies, in addition to the strong connection between EHS and abnormal findings in magnetic resonance imaging,^2^ considerable effects on memory, sleep consistency, vertigo and irregular heart rhythms have been discovered.^3^ Based on these conclusions, a few studies of the pathophysiologic mechanisms of electromagnetic radiation have been conducted. Although not yet conclusive, these studies have presented some thought‐provoking hypotheses that provide a stepping stone for other researchers seeking pharmacological materials to reverse these negative impacts. These mechanisms, above all else, produce oxidative molecules,^4^ and in the later stages disrupt catecholamine secretion, leading to accumulation of neurotoxins and subsequently disruption of the balance of the immune system,^5^ induction of cell death,^6^ changes in the expression of surface proteins,^7^ rearrangement of the genome of cells,^8^ and decreased cell motility.^9^ Following the discovery and extraction of melatonin (N‐acetyl‐5‐methoxytryptamine) from the pineal gland in 1956, extensive research into its function and structure was conducted.^10^ Even though its main source is the pineal gland, melatonin has also been extracted from different body parts such as the pancreas, retina, cerebellum, skin, ovaries etc.^11^, ^12^ For a long time it was thought that the only function of this molecule was in regulating the circadian cycle and seasonal rhythms,^13^ but extensive evidence gradually accumulated showing that melatonin also functions as an anti‐oxidant,^14^ an effective molecule in inflammatory processes,^15^, ^16^ an immunity booster^17^ and an anticancer agent.^18^, ^19^

The discovery of the antioxidant effects of melatonin in 1993^20^ coincided with a great increase in cancer research, one of the most prominent areas of research at the time, and this opened the door to further research on the molecule; out of the 22 800 subsequent studies carried out on melatonin, about 3800 of them focused on the antioxidant effects of the molecule.^21^

The general term ‘free radicals’ is assigned to molecules with one or multiple atoms with unpaired electrons, which makes them highly reactive molecules.^22^, ^23^ Extensive concrete evidence now exists of the role free radicals play in the process of developing cancer in various tissues of the body,^24^ and the pathology of the cardiovascular system,^25^, ^26^ kidneys,^27^ lungs^28^ and so on. These molecules not only come from external sources like air pollutants, tobacco, alcohol and heavy metals but also are created by endogenous processes including inflammatory processes, stress and extraneous activities.^29^ The numerous effects of these molecules and the high extent of our daily exposure to them have resulted in great efforts to find specific molecules that can pair the aforementioned electrons and protect the body against the damage caused by these radicals. These molecules are called ‘antioxidants’.^30^

Melatonin has several specific antioxidant attributes. (1) Melatonin is abundantly available in various tissues; this is required as free radicals are plentiful and have short half‐lives. (2) Melatonin's ability to react with different free radicals is a characteristic of a strong antioxidant. Melatonin has been utilized in various studies as a strong reactant with hydroxyls,^20^ alcoxyles,^31^ and peroxyls.^32^ (3) Melatonin is present in most body tissues and can be utilized as an antioxidant; the reason for this is its high lipophilicity and a good degree of water solubility. These properties allow it to cross most physiological barriers of the body effortlessly.^33^, ^34^ (4) Melatonin is abundantly available in various foods like vegetables, seeds and fruits.^35^, ^36^

It is estimated that currently around 4 800 000 000 people use cell phones around the globe.^37^ This rapid increase in the use of cell phones and other devices that emit electromagnetic radiation indicates the increasingly crucial importance of research into the effects of these rays. At present, most of the research on the effect of electromagnetic radiation is done on the gonads, and likewise research on the protective effects of melatonin has also mostly been done on the gonads of animal models.^38^, ^39^, ^40^ In addition, research on the effects of melatonin on different organs using different biomarkers and histochemical and imaging methods leads to high costs in the process of diagnosis and treatment. This limited research on specific organs of animal models and the nonhomogeneous methods of assessing melatonin's protective effects has created the need for a comprehensive and systematic study that integrates the results so far achieved.

The aim of this study is to investigate whether melatonin exerts protective effects against the electromagnetic waves emitted by mobile phones in animal models. Specifically, this research seeks to review the existing studies in this field, examining the mechanisms and outcomes of melatonin's protective effects as reported in the literature. A key focus is to determine whether the findings across studies are consistent or if significant contradictions exist, along with their potential explanations. Given the fragmented and non‐uniform results reported in this area, the purpose of this study is to systematically evaluate and synthesize the evidence regarding melatonin's protective role against the effects of mobile phone electromagnetic waves in animal models.

## 2 METHODS

### 2.1 Search resources

The systematic review was started by extracting relevant studies from search results in Google Scholar, PubMed, Embase, Scopus, Web of Science, Science Direct to determine the keywords ‘melatonin’, ‘cell phone radiations’, ‘animal model’ and expanding the search using these keywords in three stages. After a primary study of the papers over 3 weeks, the remaining papers were surveyed using the JBI checklist for systematic reviews during the course of 2 weeks and the subsequent and final surveying was completed in 2 weeks. A comprehensive search was conducted for studies published up to January 2024, without any time restrictions. This review has not been registered in any official database or system for documenting review studies.

### 2.2 Search strategy

A search strategy was implemented on PubMed using the following query: (((((((Melatonin[Title/Abstract]) AND (cell phone[Title/Abstract])) OR (Cellular Phone[Title/Abstract])) OR (Mobile Phone[Title/Abstract])) OR (Portable Cellular Phone[Title/Abstract])) AND (radiations[Title/Abstract])) AND (Models, Animal[Title/Abstract])) OR (animal model[Title/Abstract])).

This search aimed to identify studies relevant to the protective effects of melatonin against cell phone radiation in animal models.

#### 2.2.1 Inclusion criteria

Original research articles, clinical trial studies, articles with fully accessible text and data, articles written in English or providing English abstracts and studies specifically investigating the effects of cell phone radiation and melatonin in animal models.

#### 2.2.2 Exclusion criteria

Review articles, systematic reviews, and meta‐analyses, cross‐sectional and cohort studies, articles that did not provide full data, and duplicate studies.

### 2.3 The method of reviewing and collecting articles

To ensure comprehensive coverage of all relevant studies, the references of articles that met the inclusion criteria were manually reviewed. To minimize errors and maintain accuracy, all steps of the article search, study selection, quality assessment, and data extraction were conducted independently by two researchers. The identified articles from each database were imported into EndNote X8 reference management software, where duplicate entries were removed following the completion of the search process. In cases where discrepancies arose between the researchers regarding the inclusion of an article, consensus was first sought through discussion. When disagreements persisted, a third researcher was consulted to provide an independent opinion and resolve the matter, ensuring the avoidance of bias in the study selection process.

### 2.4 Checking the quality of studies

In order to check the quality of the studies included in the systematic review, the JBI Critical Appraisal Checklist for Systematic Reviews, which looks at 10 important aspects of the systematic review, was used. In order to adhere to each of the aspects examined in the reviewed articles, the scores for articles examined using this checklist ranged from 1 to 10, indicating the low to high quality of the articles in the systematic review study.

## 3 RESULTS

**FIGURE 1:**
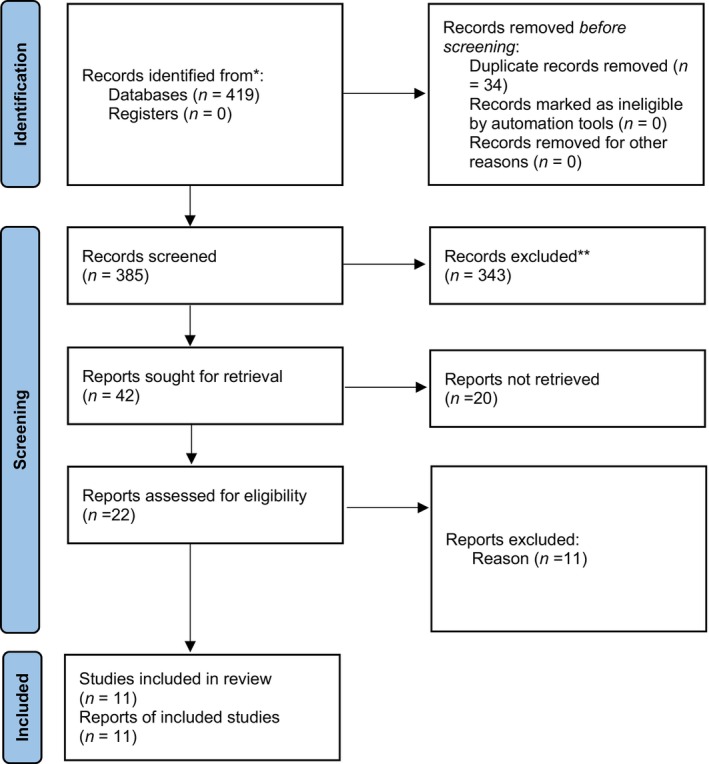
Procedures for reviewing articles based on the PRISMA flowchart.

In the initial search, 163 articles were found in the PubMed database, 19 articles were found in the WoS database, 17 articles were found in the ScienceDirect database, 18 articles were found in the Embase database, 51 articles were found in the Scopus database and 151 articles were found in the Google Scholar search engine. A total of 419 articles were obtained, and after removing 34 duplicate articles, 343 unrelated articles, and 20 articles not providing the desired information, 22 articles were included in the secondary review. After removing 11 studies at this stage due to the lack of information and the low quality of the studies, 11 studies were included in the systematic review (Figure 1). The final total of articles examined is shown in Figure 1 and Table 1.

**TABLE 1:** Information extracted from the articles reviewed in the systematic review.

Author	Year	Sampled organ	Sample Size	Sampling Method	Administered Dose	Exposure system
Köylu et al.^41^	2006	Brain	28	Simple random	100 mg/kg/day Mel	Experimental exposure system
Altun et al.^42^	2017	Brain	24	Simple random	50 mg/kg/day Mel	Experimental exposure system
Ayata et al.^43^	2004	Skin	30	Simple random	10 mg/kg/day Mel	Experimental exposure system
Kerman and Senol^44^	2012	Brain	24	Simple random	100 μg/kg/day Mel	Experimental exposure system
Seymen et al.^45^	2019	Brain	24	Simple random	10 mg/kg/day Mel	A vector signal generator
Okten et al.^46^	2005	Kidney	24	Simple random	100 μg/kg/day Mel	Experimental exposure system
Ozguner et al.^47^	2004	Skin	30	Simple random	10 mg/kg/day Mel	Experimental exposure system
Ozguner et al.^48^	2006	Eye	40	Simple random	100 μg/kg/day Mel	Experimental exposure system
Pandey and Giri^49^	2018	Testis	60	Simple random	5 mg/kg/day Mel	RFR generator
Shokri et al.^50^	2022	Testis	32	Simple random	2 mg/kg/day Mel	mobile phones
Sokolovic et al.^51^	2008	Brain	84	Simple random	2 mg/kg/day Mel	mobile phones

Köylü et al. reported that melatonin increases the mean lipid peroxidation values in the brain cortex compared to the radiation‐only group.^41^ Altun et al. demonstrated that melatonin administration increases the number of hippocampal neurons in rats exposed to cell phone radiation.^42^ Additionally, Seymen et al.^45^ observed a higher number of Purkinje neurons in rats receiving both radiation and melatonin. Ayata et al.^43^ further concluded that oxidative stress‐mediated skin damage caused by mobile phone radiation could be mitigated by melatonin treatment. A series of studies, including those by Kerman and Şenol,^44^ Ökten et al.,^46^ Özguner et al.,^46^ Shokri et al.,^50^ and Sokolovic et al.,^51^ consistently highlighted the antioxidant properties of melatonin. While radiation alone led to increased oxidative stress, these studies reported significantly higher antioxidant effects in groups receiving both radiation and melatonin across various organs. Furthermore, a qualitative study by Özguner et al.^47^ noted a lesser increase in epidermal thickness in the radiation‐plus‐melatonin group compared to radiation‐only exposure (Table 1).

Köylü et al. investigated the effects of melatonin on 28 mice, which were selected through simple random sampling. The compound was administered subcutaneously at a dosage of 100 mg/kg body weight. Melatonin was prepared in an ethanol‐saline solution at a 1:90 ratio and given 1 h prior to exposing the mice to 900 MHz radiation for a duration of 1 h. Lipid peroxidase activity was assessed using the Placer method and barbituric acid reaction. The study revealed that the brains of melatonin‐treated mice contained approximately five times more melatonin than the control group, which had a measured concentration of 40.3.^41^

In 2017, Altun et al. conducted a similar study to explore the neuroprotective effects of melatonin and omega‐3 on the hippocampus of 24 albino rats, also chosen via simple random sampling. The rats were divided into three groups: a control group (eight rats), a group exposed to 900 MHz radiation for 1 h daily, and a group exposed to the same radiation but treated with intraperitoneal melatonin at 50 mg/kg body weight. This protocol was followed for 10 days. Using the optical fraction method, the study quantified Purkinje cells in the hippocampus. The results showed that the number of Purkinje cells was comparable between the control and melatonin‐treated groups (approximately 500 cells), whereas the radiation‐only group exhibited a marked reduction to 160 cells.^52^

Kerman and Şenol also investigated the brain, employing simple random sampling to select 24 mice exposed to laboratory‐generated electromagnetic radiation. The rats were administered melatonin at 100 μg/kg, diluted in an ethanol‐saline solution (1:90 ratio), subcutaneously prior to exposure to 900 MHz radiation for 30 min. The study reported significant reductions in antioxidant enzyme activity (CAT, SOD, and GSH‐Px) in the radiation‐only group, accompanied by elevated MDA levels compared to the control group. However, the melatonin‐treated group demonstrated increased activity of antioxidant enzymes and reduced MDA levels compared to the radiation‐only group. While specific quantitative data were not disclosed, these findings underline the protective effects of melatonin against oxidative stress^44^ (Tables 2 and 3).

**TABLE 2:** Methods of melatonin administration in reviewed studies.

Author	Year	Method of melatonin administration
Köylu et al.^41^	2006	Dissolved in 1:90 ethanol/saline and injected subcutaneously (sc) daily at 17:00 h for 10 days, 1 h before each daily MW exposure period
Altun et al.^42^	2017	Injected intraperitoneally for 10 days
Ayata et al.^43^	2004	Orally for 10 days
Kerman and Senol^44^	2012	Injected and dissolved in 1:90 ethanol/saline daily at 17:00 h
Seymen et al.^45^	2019	Injected and dissolved in 10% pure ethanol for 90 days
Okten et al.^46^	2005	Dissolved in 1:90 ethanol/saline and injected subcutaneously (sc) daily at 17:00 h for 10 days, 1 h before each daily MW exposure period.
Ozguner et al.^47^	2004	Orally for 10 days
Ozguner et al.^48^	2006	Dissolved in 1:90 ethanol/saline and injected subcutaneously (sc) daily at 17:00 h for 10 days, 1 h before each daily MW exposure period
Pandey and Giri^49^	2018	Orally for 35 days
Shokri et al.^50^	2022	Orally for 30 days
Sokolovic et al.^51^	2008	Injected intraperitoneally for 10 days

**TABLE 3:** The authors, year of publication and the data extracted from these studies on three groups of animal models.

Author	Year	Received radiation only	Control group	Received both radiation and melatonin
Köylu et al.^41^	2006	Mean lipid peroxidation values of the brain cortex: 43.1	Mean lipid peroxidation values of the brain cortex: 130.1	Mean lipid peroxidation values of the brain cortex: 127.1
Altun et al.^42^	2017	Total cell number of cells in hippocampus: 500	Cell counts of pourkiing neurons: 500	Cell counts of pourkiing neurons: 1600
Ayata et al.^43^	2004	Activity of MDA: 35.45, SOD: 0.150, GSH‐px: 17.6, CAT: 0.320, HPRL: 150	Activity of MDA: 22.94, SOD: 0.273, GSH‐px: 14.37, CAT: 0.274, HPRL: 134	Activity of MDA: 23.01, SOD: 0.167, GSH‐px: 14.38 CAT: 0.141, HPRL: 91
Kerman and Senol^44^	2012	Not published	Not published	Not published
Seymen et al.^45^	2019	Cell counts of pourkiing neurons: 400	Cell counts of pourkiing neurons: 591	Cell counts of pourkiing neurons: 485
Okten et al.^46^	2005	Activity of MDA (nM g 1 wet tissue): 15.43, NAG: 0.37	Activity of MDA (nM g 1 wet tissue): 8.32, NAG: 0.08	Activity of MDA (nM g 1 wet tissue): 10.91, NAG: 0.09
Ozguner et al.^47^	2004	Changes in thickness of epidermis: 0 none, 2 mild, 2 moderate, 6 severe	Changes in thickness of epidermis: none in all	Changes in thickness of epidermis: 2 none, 2 mild, 4 moderate, 2 severe
Ozguner et al.^48^	2006	Activity of MDA: 28.36, SOD: 0.62, GSH‐px: 17.45, CAT: 1.24	Activity of MDA: 19.8, SOD: 0.88, GSH‐px: 22.52, CAT: 1.57	Activity of MDA: 20.12, SOD: 0.93, GSH‐px: 30.17, CAT: 1.61
Pandey and Giri^49^	2018	Total sperm numbers (1 000 000): 10.2, percent of abnormal sperms: 29.65	Total sperm numbers (1 000 000): 23.2, percent of abnormal sperms: 12.5	Total sperm numbers (1 000 000): 22.4, percent of abnormal sperms: 12.61
Shokri et al.^50^	2022	Activity of MDA (nM g 1 wet tissue): 4.7, GSH: 10	Activity of MDA (nM g 1 wet tissue): 2.3, GSH: 18	Activity of MDA (nM g 1 wet tissue): 4, GSH: 16
Sokolovic et al.^51^	2008	Activity of MDA (μmol/g 1 protein): 5.9, CAT: 15.1	Activity of MDA (μmol/g 1 protein): 4, CAT: 10.1	Activity of MDA (μmol/g 1 protein): 4.5, CAT: 18

In a 2019 study, Seyman et al. investigated the effects of prolonged exposure to higher‐frequency electromagnetic radiation (2100 MHz) on the brains of 24 mice. Radiation exposure was set at 30 min per day for 90 days, exceeding the intensity used in prior studies. Mice were selected through simple random sampling and received melatonin dissolved in ethanol at a dose of 10 mg/kg body weight. The researchers examined the protective effects of melatonin by estimating the number of Purkinje cells in the cerebral cortex using electron microscopy. The results revealed a marked decrease in Purkinje cell count in the radiation‐only group (400 cells) compared to the control (591 cells) and intervention (485 cells) groups^45^ (Tables 2 and 3).

Sokolovic et al.^51^ explored the protective effects of melatonin against short‐wave electromagnetic radiation. A total of 84 rats were selected via simple random sampling and injected with 2 mg of melatonin per kilogram of body weight before being exposed to 900 MHz radiation for 4 h. The study evaluated MDA activity using the Akava method^53^ and CAT activity using the Gath method.^54^ In the control group, MDA and CAT activities were recorded as 4 and 10.1, respectively. In the radiation‐only group, these activities increased to 5.9 and 15.1, respectively. Interestingly, in the intervention group, MDA activity decreased to 4.5, while CAT activity significantly increased, surpassing both other groups, with a value of 18^51^ (Tables 2 and 3).

Two additional studies focused on the effects of electromagnetic radiation on skin. Ayata et al.^43^ assessed changes in oxidizing enzyme activity in 30 laboratory rats. The animals were selected using simple random sampling and exposed to 900 MHz electromagnetic radiation for 30 min daily over 10 days. The intervention group received melatonin orally at a low dose of 10 mg/kg body weight. The activities of various enzymes, including MDA, SOD, CAT, GSH‐Px, and HPRL, were measured using established methods: MDA by Draper and Hadley,^55^ SOD by Sun,^56^ CAT by Abi,^57^ GSH‐Px by Paglia,^58^ and HPRL by Wesner.^59^ Detailed results are provided in Tables 2 and 3.

Ozguner et al. conducted a study to assess histopathological changes in the epidermal thickness of 30 rats exposed to 900 MHz radiation. The rats were chosen using simple random sampling and divided into two groups. The intervention group received 100 mg/kg of melatonin orally for 10 days, followed by daily exposure to radiation for 30 min. The study reported that in the radiation‐only group, six rats exhibited severe changes in epidermal thickness, two showed moderate changes, and two had mild changes. In contrast, the melatonin‐treated group displayed significantly fewer severe changes, with only two cases, while two rats showed no changes at all. Additionally, in the radiation‐only group, five rats exhibited severe atrophic changes in the epidermis, three showed moderate changes, and two displayed mild changes. In the intervention group, four rats exhibited no changes, four had mild changes, and two demonstrated moderate changes^47^ (Tables 2 and 3).

Okten et al. investigated the protective effects of melatonin on the kidneys of 24 rats exposed to 900 MHz electromagnetic radiation. Rats were chosen through simple random sampling and administered daily doses of 100 μg melatonin diluted in ethanol saline (1:90) for 10 days. Subsequently, the rats were exposed to radiation for 90 min. Lipid peroxidation was evaluated by measuring malondialdehyde (MDA) levels using the Draper and Hadley method.^55^ The study reported MDA activity values of 15.43 in the radiation‐only group, 10.91 in the melatonin‐treated group, and 32.8 in the control group, indicating a significant protective effect of melatonin against radiation‐induced oxidative stress^46^ (Tables 2 and 3).

In a subsequent study, Ozguner et al.^48^ explored the protective effects of melatonin and caffeic acid on the eyes of 40 rats exposed to electromagnetic radiation. The rats were randomly assigned to groups, and melatonin was administered subcutaneously at a dose of 100 μg, diluted in ethanol saline (1:90), 1 h before exposure to 900 MHz radiation for 30 min daily over 10 days. Enzyme activities for MDA, SOD, CAT, and GSH‐Px were assessed to evaluate oxidative stress. In the control group, the values were 1.57, 0.88, 19.8, and 22.52, respectively. In the melatonin‐treated group, these values increased slightly to 1.61, 0.93, 20.12, and 30.17. The radiation‐only group demonstrated reduced antioxidant enzyme activities, with values of 0.62 for SOD and 17.45 for GSH‐Px, but showed elevated levels of MDA (1.24) and CAT (28.36), further confirming melatonin's protective effects (Tables 2 and 3).

Pandey and Giri investigated the effects of melatonin on gonadal germ cells in 60 Swiss albino rats exposed to electromagnetic radiation. Using simple random sampling, the rats were divided into groups and administered 100 mg/kg of melatonin orally for 35 days, followed by exposure to radio waves for 6 h daily. The researchers measured sperm count and abnormal sperm percentages to evaluate the protective effects of melatonin. In the control group, the sperm count was 23.2 million, with 12.5% abnormal sperm. In the radiation‐only group, the sperm count dropped significantly to 10.2 million, with 29.65% abnormalities. In the intervention group treated with melatonin, the sperm count increased to 22.4 million, with only 12.6% abnormal sperm, closely resembling the control group^49^ (Tables 2 and 3).

Shokri et al.^50^ also examined the protective effects of melatonin on gonads, focusing on oxidative stress markers in rats exposed to mobile phone radiation. A total of 32 rats were selected using simple random sampling. In the intervention group, rats received oral melatonin at a dose of 2 mg/kg before being exposed to 900 MHz radiation for 4 h daily over 30 days. The activities of MDA and GSH‐Px enzymes were measured to evaluate oxidative stress. The control group had values of 2.3 and 18, respectively. In the melatonin‐treated group, MDA increased to 4, while GSH‐Px decreased to 16. In the radiation‐only group, MDA reached 4.7, and GSH‐Px activity dropped significantly to 10, indicating the mitigative effects of melatonin (Tables 2 and 3).

Finally, Sokolovic et al.^51^ conducted a study on 84 rats to investigate the protective effects of melatonin against electromagnetic radiation. Using simple random sampling, the rats were divided into groups and administered melatonin intraperitoneally at a dose of 2 mg/kg. The rats were then exposed to 900 MHz radiation for 4 h. MDA and CAT enzyme activities were measured using the Akava^53^ and Gath^54^ methods, respectively. The control group recorded MDA and CAT activity levels of 4 and 10.1, respectively. These values increased in the radiation‐only group to 5.9 and 15.1. However, in the melatonin‐treated group, MDA activity was reduced to 4.5, while CAT activity significantly increased to 18, surpassing both the control and radiation‐only groups, highlighting melatonin's antioxidant properties^51^ (Tables 2 and 3).

## 4 DISCUSSION

Many studies have provided compelling evidence of the protective effects of melatonin against the harmful impacts of electromagnetic rays emitted by mobile phones which are used increasingly and globally more and more each year.^37^ As well as being an anti‐inflammatory molecule^15^, ^16^ and an immune‐booster,^17^ melatonin is known for its potent antioxidant properties and thus has attracted the attention of many researchers. Numerous studies have examined its impact in animal models in mitigating the oxidative stress and cellular damage induced by these harmful rays in various organs such as the brain,^41^, ^42^, ^45^ the skin,^43^, ^47^ the gonads,^49^, ^50^ the eyes^48^ and the kidneys.^46^ A common theme across these studies is the mitigation of oxidative stress induced by cell phone radiation.^43^, ^46^, ^47^, ^48^, ^49^, ^50^, ^51^ Each of the organs that have been studied will be discussed separately here. For example, a study by Köylü et al. suggested a protective role of melatonin through significant reductions in lipid peroxidation in cortex and hippocampus in rat brains^41^. Another study by Sokolovic et al.^51^ showed that melatonin administration works by modulating the enzymatic activities and gene expression pathways that are involved in oxidative stress response pathways in the brain.^51^

Seymen et al.^45^ investigated the molecular pathways that were modulated by melatonin during long‐term exposure to GSM radiation. Their findings indicate that melatonin influences the NMDA‐receptor 2B/Calpain‐1/Caspase‐12 pathways and is crucial for neuronal survival and function. This alteration made by melatonin helps prevent the apoptosis process of neurons and maintains cellular integrity under electromagnetic stress.

One main finding across a number of studies is the role of melatonin in mitigating oxidative stress, a primary mechanism underlying the harmful effects of cell phone radiation. As previously mentioned, Köylu et al. reported great reductions in lipid peroxidation in the brains of rats exposed to 900 MHz microwave radiation when treated with melatonin. This suggests melatonin's capacity as a cell membrane stabilizer and preventer of oxidative damage^41^. Neurons are non‐replicating cells, and therefore protecting them from damaging mechanisms like mitochondrial DNA deletion and apoptosis is of the utmost importance. Thus research into melatonin's protective effect on the brain needs to be continued^51^.

There have been many studies on the effect of melatonin on the testis that demonstrate a possible relationship between melatonin and cell phone radiation damage to the testis that can lead to infertility.^50^ One study revealed that reactive oxygen species‐mediated oxidative stress can be caused by electromagnetic rays, resulting in damage to the DNA and germ cells, impaired spermatogenesis which leads to lower sperm counts and various defects like irregular and ineffective morphology. These damaging processes have been shown to be repressed by melatonin. Identifying the role of reactive oxygen species in the DNA damage could confirm this effect. However, the exact mechanism is not apparent and could be either through direct or indirect effects of melatonin.^49^ There is also evidence that melatonin can help increase the amount of testosterone created.^50^

A different study on the retinas of rats exposed to 900 Megahertz of radiation showed damage to the basic molecules present in the eyes, and that melatonin effectively decreased MDA and nitric oxide (NO) levels in the retina. Melatonin's protective effects against cellular oxidative damage caused by electromagnetic radiation may help treat diabetes‐related eye issues in the futures as well as other illnesses.^48^

Oktem et al.^46^ conducted a study on electromagnetic radiation damage and the protective effects of melatonin in the kidneys using an experimental exposure system. The results confirmed tissue damage from electromagnetic radiation and a protective effect of melatonin, but an interesting point was mentioned that in studies using rats, the radiation‐emitting device is most likely approximately the size of the rat's whole body, whereas if this research were to be repeated with humans using the same devices that we use daily, the smaller size of the device relative to the human body may lead to a different result.

Özguner et al. studied the effects of mobile phone radiation on skin cells and role of melatonin on those changes. The results showed that 900 MHz radiation induced increased thickness of stratum corneum, atrophy of epidermis, papillamatosis, basal cell proliferation, increased granular cell layer (hypergranulosis) in epidermis and capillary proliferation, impairment in collagen tissue distribution and separation of collagen bundles in skin cells. They also showed that treatment with a low dose of melatonin can protect against all of these changes except hypergranulosis. Radiation increases reactive oxygen species, and Özguner et al. hypothesized that external melatonin has protective effects against oxidative changes in DNA, membrane lipids, and cystolic proteins.^47^

One of the major limitations of this study was the lack of specific and uniform quantitative data in the studies included for meta‐analysis. The absence of detailed statistical information limited the accuracy and depth of the analysis, as such data could have significantly enhanced the precision and interpretation of the results. Additionally, some studies were excluded due to incomplete or insufficient data, and might have provided valuable insights had they included more comprehensive evidence and explanation. Furthermore, publication bias could not be assessed due to the limited availability of data. Finally, there are inherent challenges in generalizing findings from animal models to humans, which restricts the broader applicability of the results.

A key strength of this study was the comprehensive review of all relevant databases, ensuring that studies in this field were thoroughly identified and evaluated. This systematic and exhaustive approach enhances the reliability of the review and provides a robust foundation for understanding the protective effects of melatonin against electromagnetic radiation in animal models.

## 5 CONCLUSION

Electromagnetic radiation emitted from cell phones is proven to be harmful to various organs in animal models and the results of the current literature survey suggest that additional research should be done to achieve the best possible use of melatonin as a therapeutic agent against electromagnetic waves. Melatonin is one of the safest drugs, used for many years to help people with sleep disturbances. In order to take this research to clinical levels, it will be important to assess the primary pathophysiological theory of damage and also the mechanisms by which melatonin may protect against the specific threat, among which the most likely is by increasing the amount of antioxidants in various organs. Future reviews and interventional studies in this field should focus on determining the optimal dose and timing of melatonin administration to enhance understanding of its protective effects. Additionally, efforts should be made to develop and improve clinical trials to evaluate the benefits of melatonin in humans, enabling a deeper investigation into its potential applications and advantages in clinical settings.

## AUTHOR CONTRIBUTIONS

**Mobin Amiri:** Conceptualization; investigation; writing – original draft; writing – review and editing. **Habibolah Khazaie:** Conceptualization; supervision. **Masoud Mohammadi:** Conceptualization; investigation; methodology; supervision; writing – original draft; writing – review and editing.

## FUNDING INFORMATION

By Deputy for Research and Technology, Kermanshah University of Medical Sciences (IR) (4030031). This deputy has no role in the study process.

## CONFLICT OF INTEREST STATEMENT

The authors declare that they have no conflict of interest.

## ETHICS STATEMENT

Ethics approval was received from the ethics committee of deputy of research and technology, Kermanshah University of Medical Sciences (IR.KUMS.MED.REC.1402.283).

## CONSENT

Not applicable.

## Data Availability

Datasets are available through the corresponding author upon reasonable request.
